# Anti-complement component 5 antibody targeting MG4 domain inhibits choroidal neovascularization

**DOI:** 10.18632/oncotarget.17221

**Published:** 2017-04-19

**Authors:** Dong Hyun Jo, Jin Hyoung Kim, Wonjun Yang, Hyori Kim, Shinjae Chang, Dongjo Kim, Minseok Chang, Kihwang Lee, Junho Chung, Jeong Hun Kim

**Affiliations:** ^1^ Fight Against Angiogenesis-Related Blindness (FARB) Laboratory, Clinical Research Institute, Seoul National University Hospital, Seoul, Republic of Korea; ^2^ Department of Biomedical Sciences and Protein Metabolism, Medical Research Center, Seoul National University College of Medicine, Seoul, Republic of Korea; ^3^ Department of Biochemistry and Molecular Biology, Seoul National University College of Medicine, Seoul, Republic of Korea; ^4^ Department of Cancer Biology, Seoul National University College of Medicine, Seoul, Republic of Korea; ^5^ Cancer Research Institute, Seoul National University College of Medicine, Seoul, Republic of Korea; ^6^ Asan Institute for Life Sciences, University of Ulsan College of Medicine, Asan Medical Center, Seoul, Republic of Korea; ^7^ Biotechnology Research Institute, Celltrion, Inc., Incheon, Republic of Korea; ^8^ Department of Ophthalmology, Ajou University School of Medicine, Suwon, Republic of Korea; ^9^ Department of Ophthalmology, Seoul National University College of Medicine, Seoul, Republic of Korea

**Keywords:** age-related macular degeneration, choroidal neovascularization, complement component 5, MG4 domain, therapeutic antibody

## Abstract

Age-related macular degeneration (AMD) is one of the main causes of visual impairment in adults. Visual deterioration is more prominent in neovascular AMD with choroidal neovascularization (CNV). Clinical and postmortem studies suggested that complement system activation might induce CNV. In this study, we demonstrated that an anti-mouse complement component 5 (C5) antibody targeting MG4 domain of β chain effectively inhibited CNV which was induced by laser photocoagulation in mice. The targeted epitope of this anti-C5 antibody was different from that of currently utilized anti-C5 antibody (eculizumab) in the MG7 domain in which a single nucleotide polymorphism (R885H/C) results in poor response to eculizumab. Even with targeting MG4 domain, this anti-C5 antibody reduced production of C5a, monocyte chemoattractant protein-1 and vascular endothelial growth factor to prevent infiltration of F4/80-positive cells into CNV lesions and formation of CNV. Furthermore, anti-C5 antibody targeting MG4 domain induced no definite toxicity in normal retina. These results demonstrated that anti-C5 antibody targeting MG4 domain inhibited CNV in neovascular AMD.

## INTRODUCTION

Complement dysregulation induces the pathogenesis of age-related macular degeneration (AMD), the leading cause of blindness in adults over 50 years of age [1, 2]. One of the most important genetic risk factors in AMD is a polymorphism (Y402H) in the alternative pathway inhibitor, complement factor H [3, 4]. In addition, genetic variants in complement component 3 (C3), complement component 2 and complement factor B are also known to be associated with AMD [5, 6]. In particular, upon the activation of the complement system, activation fragments (for example, C3a and C5a) can amplify and exacerbate inflammation and tissue injury [7]. In the eye of a patient with AMD, C3a and C5a are localized to drusen, the proximity of retinal pigment epithelial (RPE) cells and Bruch's membrane [8]. In line with these results, drusen in the eyes of patients with AMD are immunopositive with complement component 5 (C5) and C5b-9 complex [9] and the C5 components are present in the drusen and RPE cells overlying or directly adjacent to the drusen [10]. In addition, increased plasma levels of Bb, C3a, C4a, and C5a are associated with AMD [11, 12] and the C5-positivity in membranes of choroidal neovascularization (CNV) is linked with the size of CNV [13]. Accordingly, various complement inhibitors were in development for the treatment of AMD [14].

In the C3-C5 axis, potential targets include individual component (for example, C3 and C5), activation fragments (for example, C3a and C5a) and C5 convertases [7]. Among them, C3 and C5 have been targets of therapeutic drugs for the treatment of AMD. POT-4 (Compstatin, Potentia) is a peptide-based drug targeting C3 [15]. On the other hand, ARC1905 (Zimura, Ophthotech), LFG316 (Novartis) and eculizumab (Soliris, Alexion) target C5 [2, 14]. Because the mechanism how C5 convertases cleave C5 remains elusive, it is important to pinpoint the binding sites of C5-targeting drugs and investigate their biological activities [16]. Furthermore, recent studies emphasized the roles of thrombin, plasmin and human neutrophil elastase other than C5 convertase in the production of activation fragments by the cleavage of C5 [17–19]. These findings added a layer of complexity in the regulation of C5 cleavage for the treatment of complement-related diseases. In addition, the polymorphisms in the target protein might be considered because they can result in poor response to antibody-based treatments, as a genetic variant in MG7 domain of C5 (R885H) attenuates clinical effects of FDA-approved eculizumab [20]. In this context, it is necessary to develop anti-C5 antibody targeting different domains of C5 with effective functional activity.

In this study, we investigated the biological activity of an antibody targeting MG4 domain of murine C5 in a murine model of laser-induced CNV. This model confers an accelerated model of neovascular AMD, demonstrating transition of phases from inflammation to neovascularization [8]. In contrast to eculizumab targeting MG7 domain which inhibits C5 cleavage by the C5 convertase, this antibody might prevent the formation of effector proteins from C5 by other proteases. Nevertheless, our anti-C5 antibody also inhibited CNV, comparable to that of BB5.1, a murine surrogate antibody to eculizumab. This anti-C5 antibody inhibits production of C5a, monocyte chemoattractant protein (MCP)-1 and vascular endothelial growth factor (VEGF) after laser photocoagulation, preventing infiltration of F4/80-positive cells into CNV lesions. Despite excellent therapeutic efficacy, anti-C5 antibody does not induce definite toxicity in normal retina. These results suggest the potential of the newly developed anti-C5 antibody in the treatment of neovascular AMD and further indications.

## RESULTS

### Development of an anti-C5 antibody targeting MG4 domain

**Figure 1:**
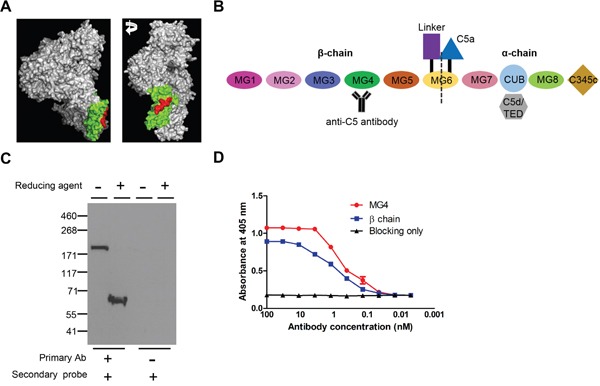
Development of anti-C5 antibody targeting MG4 domain **(A)** 3-D structure of C5 (Reference PDB 3CU7). MG4 domain and binding epitope of anti-C5 antibody were identified in green and red, respectively. **(B)** Schematic diagram of mouse C5 with anti-C5 antibody. **(C)** Western blot analyses of serum of DBA/1 mouse with anti-C5 antibody in non-reducing and reducing conditions. Representative figures from 3 independent experiments. **(D)** Binding affinity of anti-C5 antibody to MG4 domain and β-chain of mouse C5 on ELISA (*n* = 6).

An antibody targeting MG4 domain of mouse C5 was developed through immunization, phage-display, and further biopanning using recombinant β-chain and MG4 domain of mouse C5 (Figure 1A). As shown in Figure 1B, MG4 domain resides in the β-chain of C5. In non-reducing and reducing conditions, this anti-C5 antibody effectively recognized whole mouse C5 and β-chain of C5, respectively, from DBA/1 mouse sera (Figure 1C). In reducing condition, C5 is divided into α- and β-chain, which are linked to each other with disulfide bonds. Further ELISA analyses showed that anti-C5 antibody specifically bound to recombinant β-chain and MG4 domain of mouse C5 at sub-nanomolar concentrations (Figure 1D). These results demonstrated that anti-C5 antibody effectively bound to mouse C5, especially MG4 domain.

### Anti-C5 antibody targeting MG4 domain inhibits laser-induced CNV in mice

**Figure 2:**
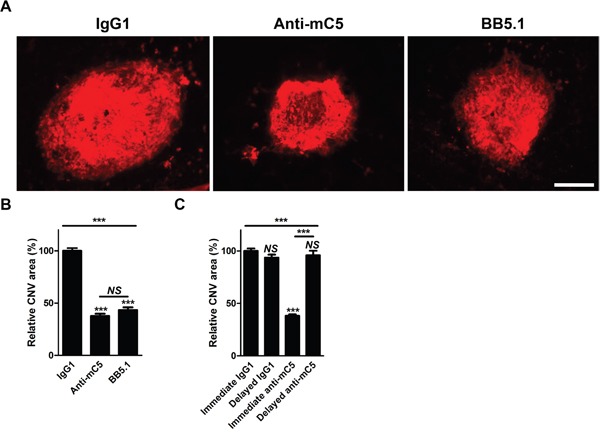
Anti-C5 antibody targeting MG4 domain inhibits laser-induced CNV in mice **(A)** Representative photographs of CNV at 7 days after laser photocoagulation and intravitreal injection of IgG1 isotype control, anti-C5 antibody, or BB5.1 demonstrated by immunostaining with isolectin B4-594. Scale bar, 200 μm. **(B)** Quantitative demonstration of relative CNV areas regarding Figure 2A (*n* = 12). **(C)** Quantitative demonstration of relative CNV areas at 7 days after laser photocoagulation. ‘Immediate’ and ‘Delayed’ indicate that antibodies were injected just after laser photocoagulation and 4 days later, respectively (*n* = 12). Anti-mC5, anti-C5 antibody; IgG1, IgG1 isotype control. NS, P-value > 0.05; ***, P-value < 0.001 (Kruskal-Wallis test with post-hoc Dunn's multiple comparison test).

There have been no reports on the functional activity of anti-C5 antibody targeting β-chain or MG4 domain. To verify the biological activity of anti-C5 antibody targeting MG4 domain, it was injected into the vitreous cavity of mice just after laser photocoagulation. In this mouse model of laser-induced CNV, anti-C5 antibody inhibited the formation of CNV, comparable to that of BB5.1, a murine surrogate antibody to eculizumab (Figure 2A and 2B). In contrast, there was no definite anti-angiogenic activity with delayed injection of anti-C5 antibody 4 days after laser photocoagulation (Figure 2C), indicating that there might be time-dependent action of anti-C5 antibody.

### Anti-C5 antibody reduces production of C5a, MCP-1 and VEGF after laser photocoagulation

**Figure 3:**
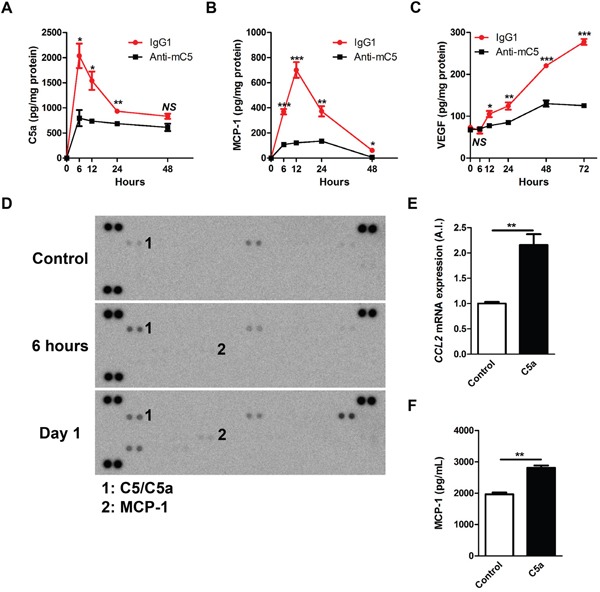
Anti-C5 antibody reduces sequential expression and secretion of C5a, MCP-1, and VEGF after laser photocoagulation **(A-C)** The levels of C5a **(A)**, MCP-1 **(B)**, and VEGF **(C)** in RPE-choroid-scleral complexes (*n* = 6). The extended versions of the graphs are provided in Supplementary Figure 1. **(D)** The patterns of multiple cytokines in RPE-choroid-scleral complexes after laser photocoagulation. Representative figures from 3 independent experiments. **(E)** Relative CCL2 mRNA expression according to the treatment with C5a in ARPE-19 cells (*n* = 6). **(F)** Amounts of VEGF in conditioned media according to the treatment with C5a (*n* = 6). Anti-mC5, anti-C5 antibody; IgG1, IgG1 isotype control. NS, P-value > 0.05; *, P-value < 0.05; **, P-value < 0.01; ***, P-value < 0.001 (Mann-Whitney U-test).

A laser-induced CNV model in mice is an accelerated model of neovascular AMD [8]. Laser photocoagulation not only induces a break in Bruch's membrane but also invokes inflammatory cascades to promote the growth of new vessels [21, 22]. As in a previous report [8], C5a was increased 6 hours after laser photocoagulation (Figure 3A and Supplementary Figure 1A). The peaks of levels of MCP-1 and VEGF were evident at 12 hours and 3 days after laser photocoagulation, respectively (Figure 3B and 3C; Supplementary Figure 1B and 1C). Cytokine arrays of 40 different cytokines also demonstrated that sequential elevation of C5a (6 hours) and MCP-1 (24 hours) in RPE-choroid-scleral complexes after laser photocoagulation (Figure 3D).

Anti-C5 antibody, when administered intravitreally just after laser photocoagulation, effectively inhibited the sequential increase in the production of C5a, MCP-1 and VEGF (Figure 3A-3C). Upon activation of the complement system, C5 is cleaved into C5a and C5b. RPE cells are known to increase the expression of various inflammatory cytokines on C5a stimulation [23, 24]. In lines with these results, C5a treatment increased the expression of *CCL2* mRNA (Figure 3E) and the secretion of MCP-1 into the media (Figure 3F) in ARPE-19 cells grown as confluent cells. These results supported the data on production of C5a and MCP-1 *in vivo*.

### Anti-C5 antibody prevents infiltration of F4/80-positive cells in CNV lesions

**Figure 4:**
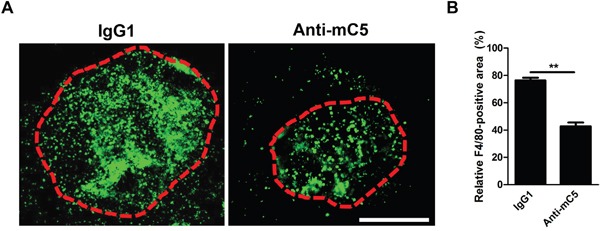
Anti-C5 antibody prevents infiltration of F4/80-positive cells in CNV lesions **(A)** Representative photographs of F4/80-positive cells in CNV lesions at 3 days after laser photocoagulation and intravitreal injection of anti-C5 antibody. Red dashed lines delineate CNV areas. Scale bar, 200 μm. **(B)** Quantitative analyses of relative F4/80-positive areas of CNV lesions (*n* = 6). Anti-mC5, anti-C5 antibody; IgG1, IgG1 isotype control. **, P-value < 0.01 (Mann-Whitney U-test).

As macrophage infiltration is observed in human CNV samples [13, 25], macrophages infiltrate into CNV lesions in a murine laser-induced CNV model and the degree of macrophage infiltration peaks at 3 days after laser photocoagulation [8, 26–28]. The infiltrated macrophages evolve to display VEGF expression [29] and are polarized to pro-angiogenic M2-type [30, 31]. Upon the treatment with anti-C5 antibody, the degree of infiltration of F4/80-positve cells was significantly decreased at 3 days after laser photocoagulation (Figure 4A and 4B). As in a previous report on the use of antibodies to C3a and C5a [8], anti-C5 antibody effectively attenuated the first (day 1) and second (day 3) increase of VEGF in a murine laser-induced CNV model (Figure 3C and Supplementary Figure 1C). The first increase in VEGF might be due to from resident cells including RPE [8], as C5a induced increased expression and secretion of VEGF in RPE cells (Supplementary Figure 2A and 2B). On the other hand, the second surge might be due to infiltrated macrophages, as they exhibit pro-angiogenic properties with VEGF expression [28–31].

### Anti-C5 antibody does not induce definite toxicity in the normal retina

**Figure 5:**
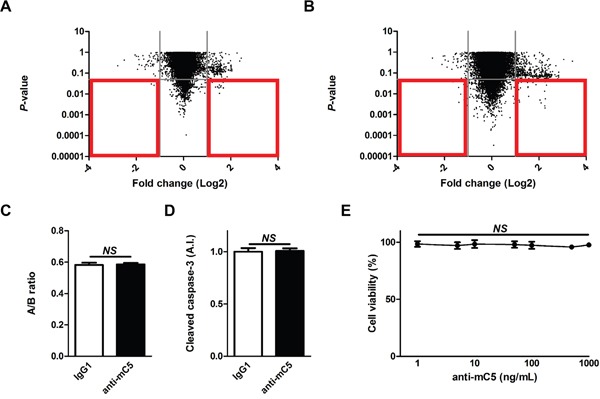
Anti-C5 antibody does not induce definite toxicity in the normal retina **(A and B)** Gene expression profiles in the retina at 7 days after intravitreal injection of (a) 1 μg and (b) 10 μg of anti-C5 antibody. Red boxes indicate differentially expressed genes of which fold changes were over 2 and P-values were less than 0.05. **(C)** The ratios (“A/B ratio”) of retinal thickness of the inner retina (from the internal limiting membrane to the inner nuclear layer, “A”) to the whole retina (from the internal limiting membrane to the outer nuclear layer, “B”) according to the treatment with IgG1 isotype control or anti-C5 antibody (*n* = 6). **(D)** Relative amounts of cleaved caspase-3 in the retina according to the treatment with IgG1 isotype control or anti-C5 antibody (*n* = 6). **(E)** Relative cell viability of bEnd.3 cells with the treatment of anti-C5 antibody at varying concentrations (*n* = 6). Anti-mC5, anti-C5 antibody; IgG1, IgG1 isotype control. NS, P-value > 0.05 (Mann-Whitney U-test in Figure 5C and 5D; Kruskal-Wallis test and post-hoc Dunn's multiple comparison test in Figure 5E).

In the development of therapeutic agents for retinal diseases, it is important to consider potential toxicity on the normal retina not to affect the vision [32, 33]. To investigate the potential toxicity of anti-C5 antibody to the normal retina, gene expression microarray was performed using the retinal samples prepared 7 days after the treatment with anti-C5 antibody at the therapeutic dose (1 μg per eye; Figure 5A and Supplementary Table 1) and 10 times the therapeutic dose (10 μg per eye; Figure 5B and Supplementary Table 2). There were 5 and 68 differentially expressed genes (fold change > 2 and *P*-value < 0.05) upon treatment with anti-C5 antibody at the therapeutic and 10 times the therapeutic doses, respectively. In addition, there were no definite changes in histologic integrity of the retinal layers, the total thickness of retinal layers (Figure 5C and Supplementary Figure 3) and apoptotic activity measured by the level of cleaved caspase-3 (Figure 5D) at 7 days after the treatment of anti-C5 antibody at 10 times the therapeutic dose. Neither cellular toxicity was observed in murine brain microvascular endothelial cells with the treatment of anti-C5 antibody at the concentrations from 1 ng/mL to 1 μg/mL in WST-1 assay (Figure 5E) and direct cell counting with methylene blue staining (data not shown).

## DISCUSSION

In this study, an anti-C5 antibody targeting MG4 domain effectively inhibited CNV in a mouse model of CNV, a well-established animal model of neovascular AMD [21]. This model demonstrates representative characteristics of neovascular AMD including the involvement of complement system, infiltration of immune cells, and CNV [8–10, 13, 25–31]. Upon laser photocoagulation to Bruch's membrane delineating RPE and choroid, the complement system is activated in response to tissue damage and exacerbates inflammation and tissue injury as in other organs [7]. In particular, as C5 and its cleaved forms of effector proteins (C5a and C5b) are expressed in human CNV [8, 9, 13] and associated with the progression of neovascular AMD [12, 13], C5a is elevated in RPE-choroid-scleral complexes [8] and the level of C5a is related with the degree of CNV formation in the laser-induced CNV model in mice [34]. In this study, we also demonstrated that C5a was elevated in RPE-choroid-scleral complexes, of which the highest value was evident at 6 hours after laser photocoagulation (Figure 3A and Supplementary Figure 1A).

**Figure 6:**
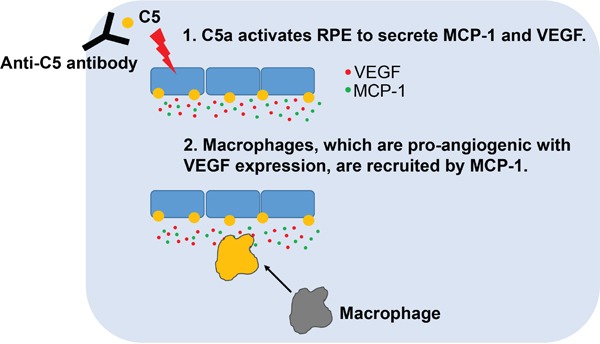
Schematic summary of the mode of action of anti-C5 antibody in the inhibition of CNV

The involvement of C5 and its cleaved effector proteins in the pathogenesis of CNV might be considered in 2 distinct phases. First, C5a directly affects RPE cells to express and secrete VEGF *in vitro* and *in vivo* [8, 35, 36]. This phenomenon was confirmed using quantitative real-time polymerase chain reaction and ELISA in this study (Supplementary Figure 2A and 2B). Second, C5a attracts immune cells including macrophages by inducing the expression and secretion of cytokines such as MCP-1 and granulocyte-macrophage colony-stimulating factor (GM-CSF) [23, 37]. In a study using a primary human RPE cell line and ARPE-19 cells which possess C5a receptors, C5a increases the expression of interleukin 6, MCP-1, and GM-CSF [23]. Similarly, C5a induced the expression and secretion of MCP-1 in ARPE-19 cells (Figure 3E and 3F) and the peak of MCP-1 level was evident at 12 hours after laser photocoagulation, following that of C5a, in the laser-induced CNV model in mice (Figure 3B and 3D; Supplementary Figure 1B). Furthermore, C5a activates the expression of intercellular adhesion molecule-1 in human choroidal and umbilical vein endothelial cells, which promotes the recruitment of monocytes/macrophages [38, 39]. Macrophages which are recruited in this way demonstrate pro-angiogenic properties, including the expression of VEGF, with the increased expression of markers of M2-type macrophages [30, 31, 40]. In this context, CNV was inhibited with C5 and C5a targeting approaches in the laser-induced CNV model in mice [8, 41–43]. In this study, anti-C5 antibody inhibited the formation of CNV with the attenuation of sequential production of C5a, MCP-1, and VEGF. Based on these results, the dynamic mechanism of action of anti-C5 antibody could be established (Figure 6). Anti-C5 antibody targeting MG4 domain bound to C5 and inhibited the cleavage of it to C5a and C5b. In this manner, the antibody prevented the activation of RPE to secrete VEGF (related with the first surge of VEGF in a laser-induced CNV model) and MCP-1, which attracts VEGF-expressing macrophages to the CNV lesions (related with the second surge of VEGF).

It is also remarkable that the inhibition of C5-mediated process was accomplished by anti-C5 antibody targeting MG4 domain in β-chain of C5. The currently utilized anti-C5 antibody in paroxysmal nocturnal hemoglobinuria, eculizumab, targets MG7 domain in α-chain, preventing the interactions between C5 and C5 convertases [16, 44, 45]. Despite its clinical success, the R885H/C mutation which is evident in 3.2-3.5% of Japanese population [20] disrupts the eculizumab epitope and is associated with poor response to eculizumab. Although the exact roles of MG4 domain in the production of active fragments are yet to be established, MG4 domain targeting might intervene the cleavage of C5 by proteases including thrombin, plasmin, and human neutrophil elastase other than C5 convertase. Inhibition via mechanically distinct strategies against C5 potentially allow fine-tuning of therapeutic effects from the regulation of C5 cleavage [16].

In conclusion, our anti-C5 antibody inhibited the formation of CNV by binding to MG4 domain of C5 and subsequently preventing C5 to be cleaved to effector proteins. Targeting the specific epitope in β-chain of C5 with sufficient biological activity confers a way to bypass resistance to ones targeting MG7 domain including Arg-885 where single nucleotide polymorphism occurs at ∼3% of the Asian population. Furthermore, with targeting to C5 in the complement system, anti-C5 antibody effectively inhibited the expression and secretion of VEGF from RPE and the infiltration of pro-angiogenic macrophages into CNV lesions, preventing the growth of CNV. This study suggested that anti-C5 antibody targeting MG4 domain might exert effective therapeutic activity against CNV and could be a valuable armamentarium against wet AMD.

## MATERIALS AND METHODS

### Development of an anti-C5 antibody targeting MG4 domain

Total RNA was prepared using TRI reagent (Invitrogen) form spleen, bone marrow and bursa of Fabricius of white leghorn chickens which had been immunized against MG4 domain and β-chain of mouse C5. First-strand cDNA was synthesized using superscript reverse transcriptase with oligo (dT) priming (Invitrogen). Using this cDNA, a phage-display library of rabbit single-chain variable fragment (scFv) was constructed using pComb3XSS phagemid vector as previously described [46, 47]. After the library construction, scFv clones were selected from the library through five rounds of biopanning as described previously [46]. For each round of biopanning, 1.5 μg of mouse MG4 domain and β-chain protein coated magnetic beads (Dynabeads M-270 Epoxy; Invitrogen) were used. The anti-mouse C5 antibody is chimeric antibody containing chicken scFv and murine IgG1 of which heavy chain and light chain were subcloned into the pCT184 and pCT146, respectively. These vectors were co-transfected to Chinese hamster ovary-K1 cells and the supernatant was subjected to protein G affinity gel chromatography for purification. This process of antibody production was supported by Celltrion.

### Western blot analysis

DBA/1 mouse sera were diluted 1/10 with PBS and electrophoresed under reducing and non-reducing conditions in a SDS-PAGE gel (NuPAGE 3-8% Tris-Acetate, Invitrogen) and transferred to a nitrocellulose membrane (Whatman) as reported previously [48]. The membrane was blocked with 5% skim milk with PBS containing 0.02% Tween-20 (Sigma) for 1 hour at room temperature (RT) and then incubated with 5 μg/ml of anti-C5 x anti-cotinine bispecific tandem scFv-human Fc fusion protein overnight at 4°C. The blots were washed with PBS containing 0.02% Tween-20 five times and incubated with 1 μg/ml of cotinine-horseradish peroxidase for 2 hours at RT. The blots were visualized as described previously [49].

### ELISA for the measurement of binding affinity of anti-C5 antibody to C5

The half-well ELISA plates (Corning) were coated with 100 ng of mouse C5 β chain and MG4 domain proteins with ckappa-tag overnight at 4°C. The wells were blocked with 150 μL of 3% BSA in PBS (w/v) for 1 hour at 37°C. The plates were washed with 150 μL of PBS containing 0.05 % Tween-20. 1 μM of anti-C5 antibody 1:3 serially diluted with 3% BSA in PBS (w/v) and incubated at 37°C for 2 hours. After washing with 150 μL of PBS containing 0.05 % Tween-20 three times, 50 μL of horseradish peroxidase-conjugated goat anti-mouse IgG (Fc) antibody (Sigma, A0168) diluted in blocking buffer (1:5,000) was added to each well and incubated for 1 hour at 37°C. After repeated washing three times, 1 μg/ml of 2, 2′-azino-bis(3-ethylbenzothiazoline-6-sulphonic acid) (Amresco) in 0.05 M citric acid buffer (pH 4.0) and 1.0 % H_2_O_2_ were added to each well, and the optical density was measured at 405 nm.

### Mice

6-week-old male C57BL/6J mice were purchased from Central Laboratory Animal and then maintained in a specific pathogen free facility in Seoul National University. All animal experiments were performed in accordance with the Association for Research in Vision and Ophthalmology statement for the use of animals in ophthalmic and vision research and all the procedures regarding animal experiments were approved by the Institutional Animal Care and Use Committee of Seoul National University.

### Laser-induced choroidal neovascularization in mice

After deep anesthesia with zolazepam plus tiletamine (3.75 mg/Kg, Virbac) and xylazyine (7.5 mg/Kg, Bayer), mice were treated with a customized laser indirect ophthalmoscope system (ILOODA) to induce the rupture of Bruch's membrane (300 μm spot size, 300 mW power, and 100 ms exposure time). After the laser photocoagulation, IgG1 isotype control (cat. no. sc3877, Santa Cruz; 1 μg/1 μL), anti-C5 antibody (1 μg/1 μL), or BB5.1 (Hycult; 1 μg/1 μL) was intravitreally administered to 12 mice per each group. For the evaluation of the effects of anti-C5 antibody on CNV, the enucleated eyes were prepared for the isolation of RPE-choroid-scleral complexes at 7 days after the laser photocoagulation. After immunostaining of RPE-choroid-scleral complexes with isolectin B4-594 (1:100; cat. no. I21413, Invitrogen), CNV was quantitatively analyzed by the measurement of the area using the ImageJ program (NIH). The absolute CNV area of IgG1-treated group (*n* = 6) was measured to be 0.15 ± 0.01 mm^2^.

### Protein preparation from RPE-choroid-scleral complex of mice

The enucleated eyes were prepared for the isolation of RPE-choroid-scleral complexes at designated time points. Then, RPE-choroid-scleral complexes from mice were put into microcentrifuge tubes 500 μL PBS containing protease inhibitor cocktail. After sonication, the tubes were centrifuged (15,000 rpm, 15 minutes) to obtain supernatants.

### ELISA for the measurement of murine C5a, MCP-1, and VEGF

ELISA was performed with corresponding kits for murine C5a (cat. no. EKU03407, Biomatik), MCP-1 (cat. no. MJE00, R&D), and VEGF (cat. no. MMV00, R&D) for the measurement of each factor in extracted proteins from the RPE-choroid-scleral complexes according to the manufacturer's instructions.

### Cytokine array

Equal amount of proteins (200 μg) prepared from the RPE-choroid-scleral complex was utilized for the measurement of the levels of cytokines using Proteome Profiler™ Mouse Cytokine Array Panel A (cat. no. ARY006, R&D), according to the manufacturer's instructions.

### Cells

ARPE-19 cells (cat. no. CRL-2302, ATCC) were maintained in DMEM:F12 supplemented with 10% fetal bovine serum and 1% penicillin-streptomycin. bEnd.3 cells (cat. no. CRL-2299, ATCC) were maintained in DMEM supplemented with 10% fetal bovine serum and 1% penicillin-streptomycin. All cells were kept in a humidified incubator at 37°C (95% air and 5% CO_2_). To monitor the responses of ARPE-19 cells to C5a, the cells grown as confluent cells were treated with human C5a (cat. no. 2037-C5-025/CF, R&D) for 72 hours after 24 hours of serum deprivation.

### ELISA for the measurement of human MCP-1 and VEGF

ELISA was performed with corresponding kits for human MCP-1 (cat. no. KHC1012, Life Technologies) and VEGF (cat. no. KHG0112, Life Technologies) for the measurement of MCP-1 and VEGF in conditioned media according to the manufacturer's instructions.

### Real-time PCR

Total RNA was isolated from cells using TRI Reagent (Molecular Research Center). Then, the cDNA was prepared with High Capacity RNA-to-cDNA kit (Life Technologies). Real-time PCR was performed with TaqMan® fast advanced master mix (Life Technologies) and Gene Expression Assays (cat. no. 4331182, Life Technologies). Product IDs of specific Gene Expression Assays for *CCL2*, *VEGF*, *GAPDH*, and *18S* were Hs00234140_m1, Hs00900055_m1, Hs99999905_m1, and Hs99999901_s1. Real-time PCR was performed using StepOnePlus RT-PCR System (Life Technologis) and the results were analyzed with accompanying StepOne Software (ver. 2.2). All procedures were in accordance with MIQE guidelines.

### Gene expression microarray

Mice (*n* = 12) were treated with intravitreal injection of IgG1 isotype (1 μg/1 μL PBS) or anti-C5 antibody (1 or 10 μg/1 μL PBS). At 7 days after the injection, retinas were prepared from the enucleated eyes. Four retinas from 4 mice were put into a microcentrifuge tube for further analyses. For each condition, 3 biological replicates were prepared including 12 retinas. Total RNA was isolated using TRI Reagent (Molecular Research Center). The RNA quality was assessed by Agilent 2100 bioanalyzer using the RNA 6000 Nano Chip (Agilent) and the quantity was determined by ND-2000 Spectrophotometer (Thermo). Equal amounts of total RNA (300 ng) from each sample was converted to double-stranded cDNA. Using a random hexamer incorporating a T7 promoter, amplified RNA (cRNA) was generated from the double-stranded cDNA template though an *in-vitro* transcription reaction and purified with the Affymetrix sample cleanup module. cDNA was regenerated through a random-primed reverse transcription using a dNTP mix containing dUTP. The cDNA was then fragmented by uracil-DNA-glycosylase and apurinic/apyrimidinic endonuclease 1 and end-labeled by terminal transferase reaction incorporating a biotinylated dideoxynucleotide. Fragmented end-labeled cDNA was hybridized to the GeneChip® Mouse Gene 1.0 ST arrays for 16 hours at 45°C and 60 rpm as described in the GeneChip Whole Transcript Sense Target Labeling Assay Manual (Affymetrix). After hybridization, the chips were stained and washed in a Genechip Fluidics Station 450 (Affymetrix) and scanned by using a GeneChip® Scanner 3000 7G (Affymetrix). The image data was extracted through Affymetrix Command Console software1.1. Expression data were generated by Affymetrix Expression Console software version1.1. For the normalization, Robust Multi-Average algorithm implemented in Affymetrix Expression Console software was used. All experiments were performed triplicate. The data discussed in this publication have been deposited in NCBI's Gene Expression Omnibus [50] and are accessible through GEO Series accession number GSE95742 (https://www.ncbi.nlm.nih.gov/geo/query/acc.cgi?acc=GSE95742).

### Histologic analysis

Mice were treated with anti-C5 antibody (10 μg/1 μL PBS) via intravitreal administration (*n* = 6). At 7 days after the injection, the enucleated eyes were fixed in 4% paraformaldehyde and embedded in paraffin. Then, 4-μm-thickness paraffin sections were deparaffinized and hydrated by sequential immersion in xylene substitute and graded ethyl alcohol solutions. To investigate the histologic toxicity of anti-C5 antibody, H&E-stained slides were evaluated to measure the ratio of the retinal thickness from the internal limiting membrane to the inner nuclear layer to that from the internal limiting membrane to the outer nuclear layer [51].

### ELISA for the measurement of cleaved caspase-3

ELISA was performed with a kit (cat. no. DYC835-2, R&D) for the measurement of cleaved caspase-3 in extracted proteins from the retina according to the manufacturer's instructions.

### Cell viability assay

bEnd.3 cells in 96-well plates were treated with varying concentrations of anti-C5 antibody (1-1,000 ng/mL). At 48 hours after the treatment, the reagent from EZ-Cytox Cell Viability Assay Kit (Itsbio) was applied to each well. After 2 hours of additional incubation, the absorbance at 450 nm was measured using the microplate reader. These results were confirmed by direct cell counting with methylene blue staining.

### Statistical analysis

All statistical analyses were performed using GraphPad Prism 5 (GraphPad). Mean values and SEM were shown in figures. Specific *P*-values and statistical methods were provided in figure legends.

## SUPPLEMENTARY FIGURES AND TABLES




