# Supplementary material for: Anti-complement component 5 antibody targeting MG4 domain inhibits choroidal neovascularization
Source: Oncotarget. 2017 Apr 19;8(28):45506–16. doi: 10.18632/oncotarget.17221 (PMC5542204; doi:10.18632/oncotarget.17221)
Supplement: Supplementary file 1 [file oncotarget-08-45506-s001.pdf]

Anti-complement component 5 antibody targeting MG4 domain inhibits choroidal neovascularization

SUPPLEMENTARY FIGURES AND TABLES

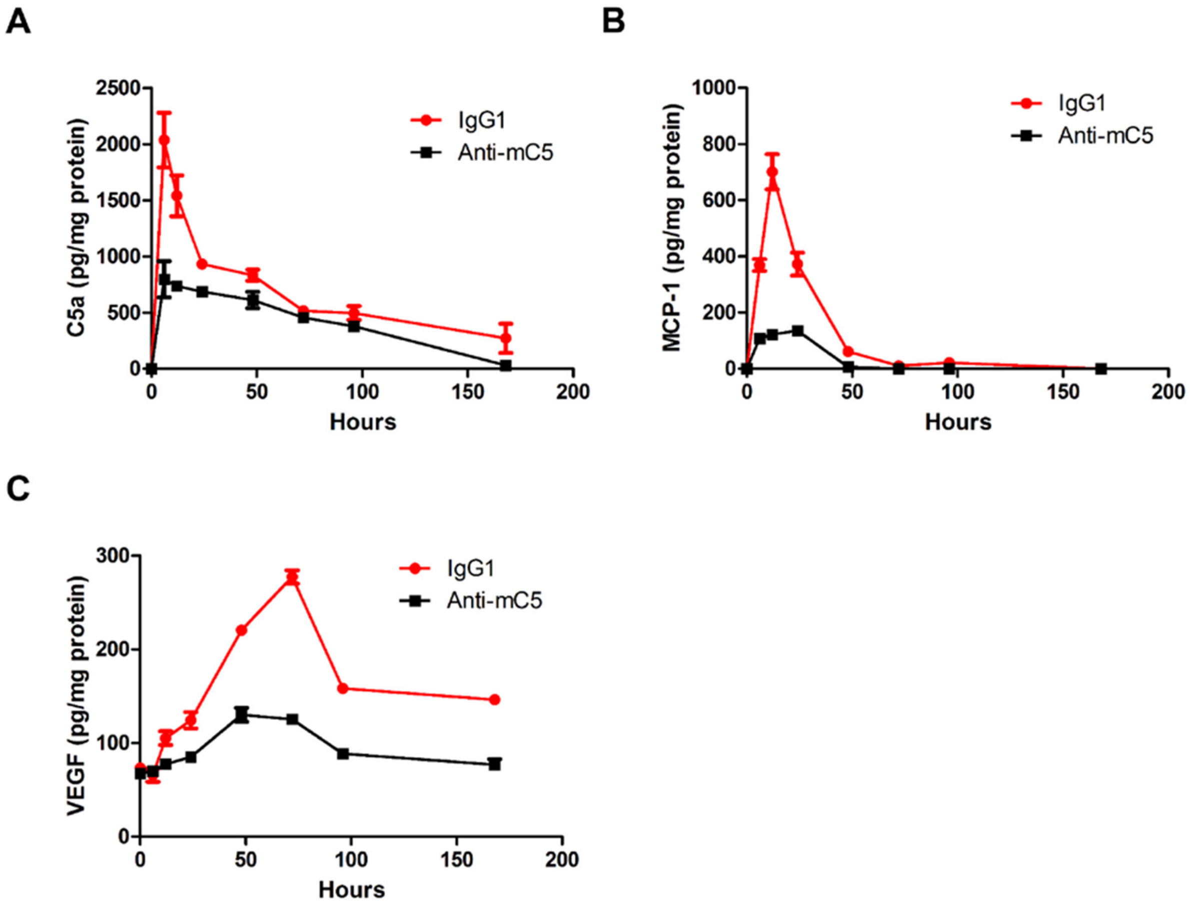

Supplementary Figure 1: Anti-C5 antibody reduces production of C5a, MCP-1, and VEGF after laser photocoagulation. **A-C.** The levels of C5a (A), MCP-1 (B), and VEGF (C) in RPE-choroid-scleral complexes. Anti-mC5, anti-C5 antibody; IgG1, IgG1 isotype control.

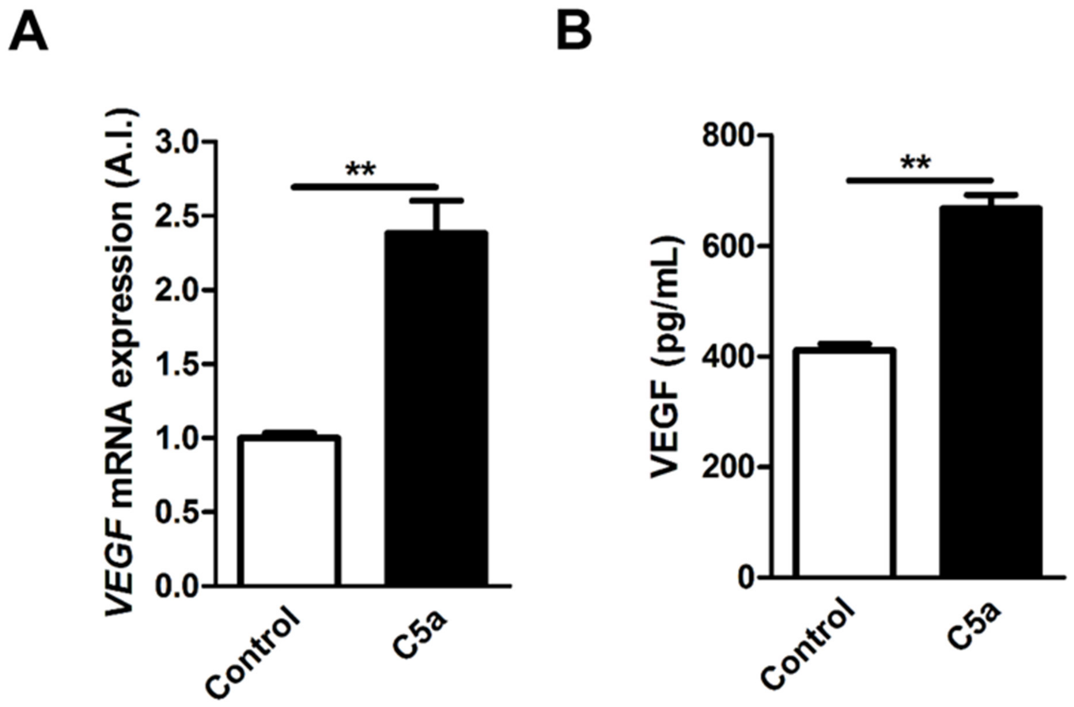

Supplementary Figure 2: C5a promotes the expression and secretion of VEGF in ARPE-19 cells. (A) Relative VEGF mRNA expression according to the treatment with C5a. **(B)** Amounts of VEGF in conditioned media according to the treatment with C5a. **, *P*-value < 0.01 (Mann-Whitney U-test).

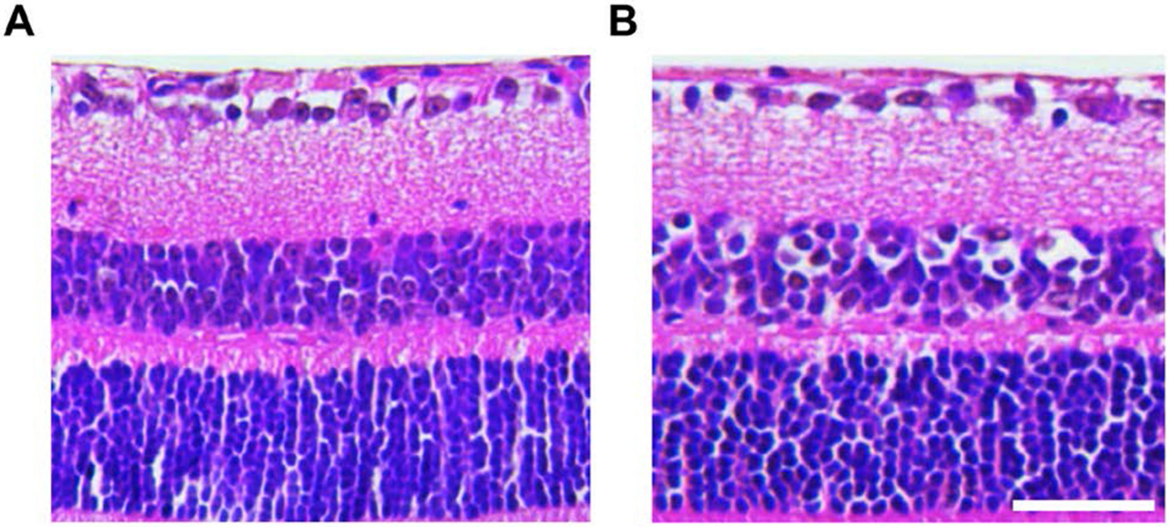

Supplementary Figure 3: Anti-C5 antibody does not induce definite histologic toxicity in the normal retina. (**A** and **B**) Representative H&E images of mice treated with (A) IgG1 isotype control and (B) anti-C5 antibody (10 μg/eye; 10 times the therapeutic dose in this study). Scale bar, 50 μm.

Supplementary Table 1: The list of differentially expressed genes (total 5 genes; > 2 fold and *P*-value < 0.05) in the retina by intravitreal injection of anti-C5 antibody (1 µg/eye; the therapeutic dose in this study)

Gene symbol	Fold change	*P*-value	Gene description
Gm20738	2.003	0.04321685	predicted gene, 20738
Gm20830	3.010	0.03594047	predicted gene, 20830
Mir466g	0.403	0.01616836	microRNA 466g
Mir669n	0.384	0.01081764	microRNA 669n
Vmn1r157	2.548	0.02041716	vomeronasal 1 receptor 157

Supplementary Table 2: The list of differentially expressed genes (total: 68 genes; > 2 fold and *P*-value < 0.05) in the retina by intravitreal injection of anti-C5 antibody (10 µg/eye; 10 times the therapeutic dose in this study)

See Supplementary File 1
