# Supplementary material for: Anti-complement component 5 antibody targeting MG4 domain inhibits choroidal neovascularization
Source: Oncotarget. 2017 Apr 19;8(28):45506–16. doi: 10.18632/oncotarget.17221 (PMC5542204; doi:10.18632/oncotarget.17221)
Supplement: Supplementary file 2 [file oncotarget-08-45506-s002.doc]

**Supporting Table 2.** The list of differentially expressed genes (total: 68 genes; > 2 fold and *P*-value < 0.05) in the retina by intravitreal injection of anti-C5 antibody (10 μg/eye; 10 times the therapeutic dose in this study).

| **Gene symbol** | **Fold change** | ***P*-value** | **Gene description** |
| --- | --- | --- | --- |
| Astx | 4.816 | 0.0499458 | amplified spermatogenic transcripts X encoded |
| Gm10230 | 3.347 | 0.04674871 | predicted gene 10230 |
| Gm10400 | 0.471 | 0.01346121 | predicted gene 10400 |
| Gm10717 | 2.449 | 0.0416555 | predicted gene 10717 |
| Gm10718 | 2.336 | 0.04384291 | predicted gene 10718 |
| Gm10719 | 2.322 | 0.04300439 | predicted gene 10719 |
| Gm11105 | 2.090 | 0.04593168 | predicted gene 11105 |
| Gm12735 | 2.983 | 0.03578978 | predicted gene 12735 |
| Gm16404 | 2.326 | 0.04239244 | predicted gene 16404 |
| Gm16583 | 2.116 | 0.01926463 | predicted gene 16583 |
| Gm2030 | 5.970 | 0.04869368 | predicted gene 2030 |
| Gm20738 | 7.131 | 0.04242767 | predicted gene 20738 |
| Gm20815 | 5.917 | 0.04675261 | predicted gene 20815 |
| Gm20831 | 3.767 | 0.04710497 | predicted gene 20831 |
| Gm21732 | 5.049 | 0.04393758 | predicted gene 21732 |
| Gm21882 | 3.608 | 0.00265174 | predicted gene 21882 |
| Gm21943 | 5.455 | 0.04920634 | predicted gene 21943 |
| Gm23209 | 0.429 | 0.01326047 | predicted gene 23209 |
| Gm23422 | 0.465 | 0.00157769 | predicted gene 23422 |
| Gm23505 | 2.750 | 0.04366799 | predicted gene 23505 |
| Gm23752 | 0.473 | 0.00583173 | predicted gene 23752 |
| Gm24089 | 2.681 | 0.04895648 | predicted gene 24089 |
| Gm24114 | 0.453 | 0.01541513 | predicted gene 24114 |
| Gm24342 | 0.481 | 0.03562895 | predicted gene 24342 |
| Gm24564 | 0.403 | 8.35E-04 | predicted gene 24564 |
| Gm24938 | 2.098 | 0.0074733 | predicted gene 24938 |
| Gm25224 | 0.487 | 0.0361432 | predicted gene 25224 |
| Gm25568 | 0.279 | 0.01034816 | predicted gene 25568 |
| Gm25599 | 2.890 | 0.00854563 | predicted gene 25599 |
| Gm26065 | 2.871 | 0.00743723 | predicted gene 26065 |
| Gm26355 | 0.408 | 0.00253914 | predicted gene 26355 |
| Gm26818 | 2.231 | 0.02728685 | predicted gene 26818 |
| Gm7609 | 2.814 | 0.022004 | predicted pseudogene 7609 |
| Gm8020 | 2.298 | 0.01026265 | predicted gene 8020 |
| Gm9912 | 0.495 | 0.01668307 | predicted gene 9912 |
| Gmcl1l | 4.760 | 0.01535706 | germ cell-less homolog 1 (Drosophila)-like |
| Hamp | 2.054 | 0.03906116 | hepcidin antimicrobial peptide |
| LOC102631913 | 3.998 | 0.03801394 | Y-linked testis-specific protein 1-like |
| LOC102636963 | 2.241 | 0.01766247 | keratin-associated protein 20-2-like |
| LOC102638087 | 2.239 | 0.03720673 | keratin-associated protein 20-2-like |
| LOC102642160 | 0.331 | 0.01087588 | uncharacterized LOC102642160 |
| Mir181a-1 | 0.444 | 0.00698111 | microRNA 181a-1 |
| Mir297a-4 | 0.475 | 0.0267968 | microRNA 297a-4 |
| Mir300 | 0.452 | 0.00494732 | microRNA 300 |
| Mir370 | 0.486 | 0.00964984 | microRNA 370 |
| Mir466g | 0.382 | 0.01895864 | microRNA 466g |
| Mir466p | 0.499 | 0.02403109 | microRNA 466p |
| Mir493 | 0.496 | 0.00609135 | microRNA 493 |
| Mir494 | 0.478 | 0.02923427 | microRNA 494 |
| Mir669a-3 | 0.484 | 0.01880888 | microRNA 669a-3 |
| Mir669m-1 | 0.460 | 0.00777609 | microRNA 669m-1 |
| Mir669o | 0.378 | 0.00391651 | microRNA 669o |
| Mir690 | 0.474 | 0.00981349 | microRNA 690 |
| Mir701 | 0.410 | 0.00579938 | microRNA 701 |
| Mir876 | 0.481 | 0.03202348 | microRNA 876 |
| Mirlet7c-2 | 0.439 | 0.01128627 | microRNA let7c-2 |
| Mup1 | 2.355 | 0.00644229 | major urinary protein 1 |
| Mup10 | 2.154 | 0.00411493 | major urinary protein 10 |
| Mup13 | 2.053 | 0.0158864 | major urinary protein 13 |
| Mup2 | 2.150 | 0.00764982 | major urinary protein 2 |
| Olfr1437 | 0.479 | 0.00296858 | olfactory receptor 1437 |
| Olfr1501 | 3.044 | 0.03605954 | olfactory receptor 1501 |
| Olfr1502 | 0.415 | 4.27E-04 | olfactory receptor 1502 |
| Olfr310 | 0.417 | 0.00583803 | olfactory receptor 310 |
| Rbmy | 2.138 | 0.00404275 | RNA binding motif protein, Y chromosome |
| Sly | 2.465 | 0.03990894 | Sycp3 like Y-linked |
| Speer4e | 10.668 | 0.04574269 | spermatogenesis associated glutamate (E)-rich protein 4e |
| Trbj1-7 | 3.085 | 0.01137457 | T cell receptor beta joining 1-7 |
